# Identification of a potential internalization or endocytic compartment-targeting signal in the C-terminal tail of the tetraspanin protein TSP-12

**DOI:** 10.17912/micropub.biology.002083

**Published:** 2026-04-24

**Authors:** Zhiyu Liu, Byron Williams, Jun Liu

**Affiliations:** 1 Department of Molecular Biology and Genetics, Cornell University

## Abstract

Tetraspanins are unique yet highly conserved four-pass transmembrane proteins that play important roles in diverse biological processes. The *C. elegans* tetraspanin protein TSP-12 is localized both at the cell surface and in multiple intracellular compartments, including different endosomal vesicles and the Golgi. In this study, we found that mutating key residues in the short C-terminal intracellular domain of TSP-12 (ILxxxxxWYY) led to an increase of cell surface localization of TSP-12 without affecting its localization in the ER or Golgi. These observations suggest that the ILxxxxxWYY motif in TSP-12 represents a potential internalization or endocytic compartment-targeting signal.

**Figure 1. Imaging and western blotting results showing that mutations in the C-terminal tail of TSP-12 lead to increased localization of TSP-12 at or near the cell surface:** **A** ) Predicted structure of TSP-12 by AlphaFold3. Both the N- (green) and C- (yellow) termini of TSP-12 are localized to the cytoplasmic side. Two extracellular domains, LEL (large extracellular loop) and SEL (small extracellular loop), are shown in orange and dark blue, respectively. Four transmembrane (TM) α-helices are colored in royal blue. The putative sorting signals in the C-terminus are labeled in red (arrows). **B** ) The amino acid sequence of TSP-12 with the transmembrane domains shown in royal blue and the 5 amino acids, IL and WYY, shown in red. **C** ) The C-terminal tail amino acid sequence of TSP-12 showing mutation of IL and WYY in wild-type (WT) to AA and AAA (red) in TSP-12(5A mut). D) Western blot showing GFP::TSP-12 protein and GFP::TSP-12(5A mut) protein probed with anti-GFP antibodies. CGC1 worms do not express GFP::TSP-12 and are used as a negative control. 40 μg total protein (equivalent to ~200 young adult hermaphrodites) are used in each lane. Samples of each genotype were run in duplicate lanes. * marks non-specific, anti-GFP antibody cross-reacting band. MW, molecular weight ladder. Actin is used as the protein loading control. Compared to WT, the amount of the ~55kDa band is decreased while levels of ~60kDa bands are increased for GFP::TSP-12 in *tsp-12(5A mut)* mutant. In addition, the GFP::TSP-12(5A mut) protein migrates slightly faster than the wild-type protein, as compared to the mobility of the non-specific (*) and actin bands. **E-H** ) and **M-P** ) Airyscan confocal images showing localization of GFP::TSP-12 (E-H), SUP-17::GFP (M-P), with the ER and *cis* -Golgi marker mCherry::RER-1 (E'-P') in early embryos (E-F, M-N) and proximal oocytes (G-H, O-P). Scale bar, 10μm. **I-L) and Q-T** ) Quantification of normalized cell surface over cytoplasmic localization of TSP-12 (I, K) and SUP-17 (Q, S), with the respective wild-type value set as 1.00, and the colocalization of TSP-12 (J, L) or SUP-17 (R, T) with RER-1. Pearson's coefficients indicate the correlated relationship between GFP::TSP-12 or SUP-17::GFP and mCherry::RER-1. Manders' M1 indicates the fraction of GFP::TSP-12 or SUP-17::GFP that is colocalized with mCherry::RER-1. Manders' M2 indicates the fraction of mCherry::RER-1 that is colocalized with GFP::TSP-12 or SUP-17::GFP. Manders' Colocalization Coefficients (M1 and M2) were determined by specifically quantifying the punctate signal of mCherry::RER-1, which labels the *cis* -Golgi. Statistical analysis was conducted using R by comparing 5A mutant embryos/germlines with WT embryos/germlines using ANOVA followed by Tukey HSD. ^***^ *P* < 0.001; ^*^ 0.01< *P* < 0.05; n.s., no significant difference.

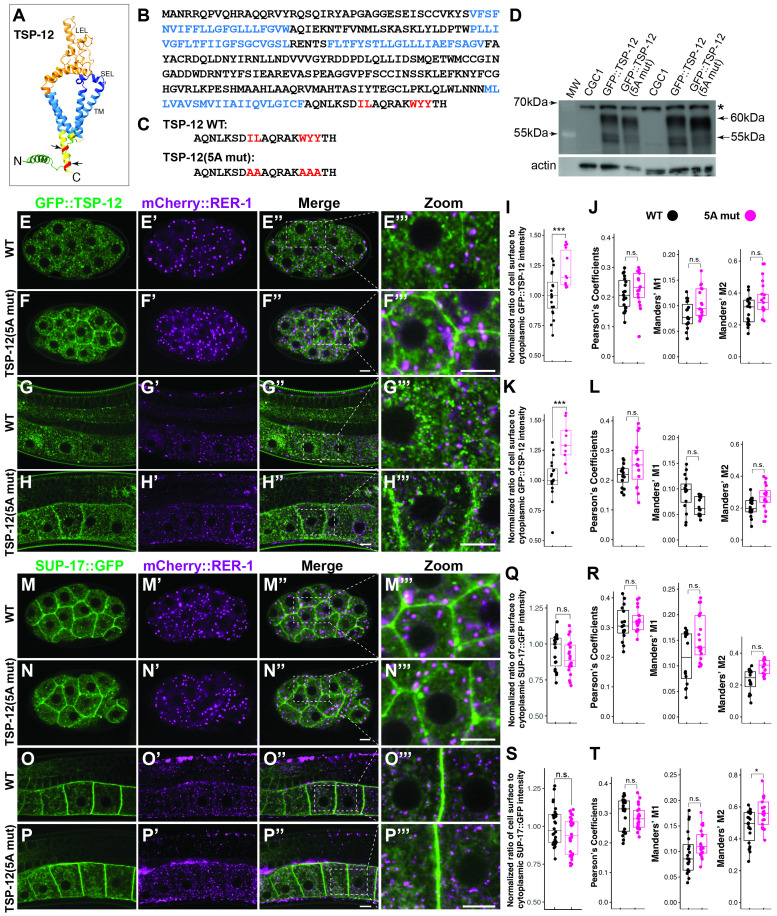



## Description

Transmembrane proteins targeted for the secretory pathway, cell surface, or endosomal compartments involve precise sorting mechanisms. Nascent cargo proteins undergo quality control within the endoplasmic reticulum (ER) to ensure correct folding and modification. Following this, export-competent proteins accumulate at ER export sites (ERES) and traverse the ER-Golgi interface via coat protein complex II (COPII)-coated vesicles (Farhan et al., 2025; Watson et al., 2025). The cargo proteins then undergo anterograde transport through the Golgi, being further modified via glycosylation before reaching the *trans* -Golgi network (TGN). As the primary sorting hub, the TGN distributes cargo proteins to the plasma membrane, secretory granules, or the endolysosomal system. Meanwhile, retrograde trafficking retrieves escaped ER proteins or recycles Golgi-resident proteins (Dancourt and Barlowe 2010; Downes and Zanetti 2025). A number of targeting signals on cargo proteins have been identified to serve either as export signals, retention or retrieval motifs, or internalization and/or endolysosomal sorting signals (Dell et al., 2019). For example, cytosolic export signals, such as di-acidic [e.g., (D/E)x(D/E), x stands for any amino acid], di-hydrophobic(e.g., LL, IL), or di-aromatic motifs (e.g., FF, YY, LxxLE), are known to recruit the Sec23/Sec24 subunits of the COPII complex at ER export sites for efficient ER exit of cargo proteins (Barlowe 2003; Malhotra 2024). Conversely, cytosolic KKxx motifs (which recruit COPI for ER retrieval), luminal KDEL motifs (which are recognized by the KDEL receptor), and Golgi-retention signals such as the Vps74/GOLPH3-binding motif [(F/L)(L/I/V)xx(R/K)] (Gomez-Navarro and Miller 2016; Tu et al., 2008), serve as retention or retrieval signals for maintaining ER or Golgi localization of cargo proteins (Watson et al., 2025). Additionally, transmembrane proteins can be recycled through a cycle of endocytosis, sorting in early endosomes, and recycling back to the cell surface via either recycling endosomes or the retromer pathways. Motifs such as NPxY, YxxØ (Ø: an amino acid with a bulky hydrophobic side chain), [DE]xxxL[LI], and DxxLL have been found to serve either as internalization or endolysosomal targeting signals (Bonifacino and Traub 2003; Hsu et al., 2012; Kelly and Owen 2011).

We have been studying a highly conserved tetraspanin protein TSP-12 in *C. elegans* ( Figure 1A ). TSP-12 and its paralog TSP-14 function redundantly to regulate both Notch signaling and BMP signaling (Dunn et al., 2010; Liu et al., 2020; Wang et al., 2017). Our previous work has shown that TSP-12 is localized to multiple cellular compartments, including the cell surface, the Golgi, and early, late and recycling endosomes (Liu et al., 2020; Liu et al., 2026; Wang et al., 2017). When analyzing the protein sequence of TSP-12 , we found two putative COPII (ER to Golgi) sorting motifs in the C-terminal tail of TSP-12 , IL and WYY (ILxxxxxWYY, Figure 1B ). To test the importance of these motifs for the localization and function of TSP-12 , we first used CRISPR/Cas9 to change the IL and the WYY residues to alanines in an endogenous GFP::TSP-12 background, generating the "5A mutant" (abbreviated 5A mut; Figure 1C ). The “5A mutant” animals do not have any detectable phenotypes. To our surprise, in both early embryos and proximal oocytes, GFP::TSP-12(5A mut) does not appear to accumulate in either the ER or the *cis* -Golgi compared to wild-type (WT) GFP::TSP-12. Instead, there is increased localization of GFP::TSP-12(5A mut) at or near the cell surface (Figure1E-L). These observations suggest that the ILxxxxxWYY motif is unlikely to be a sorting motif for the ER-to-Golgi trafficking of TSP-12 .

We have previously shown that on western blots, WT GFP::TSP-12 can be detected as two major bands at around 60kDa, representing both glycosylated and un-glycosylated forms of GFP::TSP-12, as well as a band near 55kDa (Liu et al., 2026). As shown in Figure 1D, we observed a decrease in the amount of the ~55kDa band and an increase in the amount and a downward mobility shift of the 60kDa bands in the GFP::TSP-12(5A mut) lane compared to the WT GFP::TSP-12 lane. The downward mobility shift may indicate differential glycosylation in the 5A mut, or may be simply due to the amino acid changes altering migration of the mutant protein in the gel. Because the ~55kDa band is absent in *sup-17 (ts)* mutants at the restrictive temperature when GFP::TSP-12 protein is accumulated in the ER, we speculated previously that the ~55kDa band may represent a cleaved form of GFP::TSP-12 produced after the protein has trafficked past the Golgi, possibly due to endocytic processing (Liu et al., 2026). The reduced amount of this ~55kDa band, along with increased level and near cell surface localization of GFP::TSP-12(5A mut) is consistent with this hypothesis, suggesting that the increased GFP::TSP-12(5A mut) protein near the cell surface might be a consequence of the GFP::TSP-12(5A mut) mutant protein unable to be internalized or to be sorted to various endosomal compartments.

TSP-12 and SUP-17 /ADAM10 are known to exhibit cell type-specific interdependence in their trafficking through the Golgi (Liu et al., 2026). To determine whether increased localization of TSP-12 (5A mut) near the cell surface affects SUP-17 localization, we next generated the TSP-12 (5A mut) mutation in the untagged TSP-12 background and examined SUP-17::GFP localization. As shown in Figure 1(M-T), the TSP-12 (5A mut) mutation did not significantly alter the cell surface or intracellular localization of SUP-17::GFP in either embryos or proximal oocytes.

In summary, we have identified a putative internalization or endocytic compartment-targeting signal in the C-terminal tail of the tetraspanin protein TSP-12 . The ILxxxxxWYY motif has similarities to, but is not identical to, previously identified internalization or endolysosomal targeting motifs (NPxY, YxxØ, [DE]xxxL[LI], and DxxLL). Future work will be needed to identify the specific coat protein complexes that mediate TSP-12 internalization or targeting to the endocytic compartment.

## Methods

All *C. elegans* strains used in this study were derived from CGC1 (Ichikawa et al., 2025), and were maintained at 20°C (unless otherwise noted *). C. elegans* strains used and generated in this study are summarized under Reagents.

**CRISPR/Cas9 experiments**

CRISPR/Cas9 experiments were conducted by following the protocol described in (Liu et al., 2026). The genotypes of the final strains were confirmed through Sanger sequencing. Oligonucleotides used in this study are summarized under Reagents.

**Live imaging and image analysis**

Imaging experiments and subsequent image analysis were conducted by following the protocol described in (Liu et al., 2026).

**Western blot analysis**

Western blot experiments were conducted by following the protocol described in (Liu et al., 2026). Goat anti-GFP (Rockland Immunochemicals, Item No. 600-101-215) (1:3,000), mouse anti-actin IgM (JLA20, Developmental Studies Hybridoma Bank) (1:2,000), HRP-conjugated donkey anti-goat IgG (Jackson Immunoresearch) (1:10,000), and IRdye 800CW-conjugated goat anti-mouse IgM (Licor) (1:10,000) were used.

## Reagents

**Strains used in this study:**

**Table:** 

**STRAIN**	**GENOTYPE**
LW3705	*sup-17 ( jj98 [SUP-17::GFP]) *
LW4453	*tsp-12 ( jj181 [GFP::3xFLAG::TSP-12]) *
GK171 (LW7015)	*dkIs91 (pie-1p::mCherry::RER-1)* [Gift from Ken Sato (Sakaguchi et al., 2015)]
LW7039	*sup-17 ( jj98 [SUP-17::GFP]); dkIs91 (pie-1p::mCherry::RER-1) isolate #1 *
LW7040	*sup-17 ( jj98 [SUP-17::GFP]); dkIs91 (pie-1p::mCherry::RER-1) isolate #2 *
LW7178	*sup-17 ( jj98 [SUP-17::GFP]); tsp-12 (jj551[5A mut]); dkIs91 (pie-1p::mCherry::RER-1) *
LW7179	*sup-17 ( jj98 [SUP-17::GFP]); tsp-12 (jj552[5A mut]); dkIs91 (pie-1p::mCherry::RER-1) *
LW7037	*tsp-12 ( jj181 [GFP::3xFLAG::TSP-12]); dkIs91 (pie-1p::mCherry::RER-1) isolate #1 *
LW7038	*tsp-12 ( jj181 [GFP::3xFLAG::TSP-12]); dkIs91 (pie-1p::mCherry::RER-1) isolate #2 *
LW7180	*tsp-12 ( jj181 jj553[GFP::3xFLAG::TSP-12 (5A mut]]); dkIs91 (pie-1p::mCherry::RER-1) *
LW7181	*tsp-12 ( jj181 jj554[GFP::3xFLAG::TSP-12 (5A mut]); dkIs91 (pie-1p::mCherry::RER-1) *

**&nbsp;**

**Oligonucleotides used in this study:**

**Table:** 

**Oligonucleotides used for genotyping**		
***sup-17 ( jj98 [SUP-17::GFP]) ***		LW27 (ATCCAGGATCAGCAGCTGTA, R)
**&nbsp;**		LW30 (AAGTGTTGCGCTGTACACAC, F)
**&nbsp;**		JKL-269 (AAGCGTTCAACTAGCAGACC, F)
***tsp-12 ( jj181 [GFP::3xFLAG::TSP-12]) ***		ZL431 (GGAGAATCTGTACTTTCAATCCGG, F)
**&nbsp;**		ZL374 (CCCTCTGCTGTGCTCTGTGTTGC, R)
**&nbsp;**		ZL375 (CTGAAGGGGAACATTTCTTTCCTGG, F)
***tsp-12 (jj551/jj552/jj553/jj554[ TSP-12 (5A mut)]) ***		
Genotyping		ZL1045 (GTGACCGGCATAGGCTGTG, R)
&nbsp;		ZL1046 (GTAGCCGTTTCGATGGTTATTATTGC, F)
&nbsp;		ZL1060 (TCAACGGGCAAAATGGTATTAT, F)
Sequencing		ZL1024 (CAATAGAAGCCAGAGAAGTGGC, F)
**&nbsp;**		ZL1045 (GTGACCGGCATAGGCTGTG, R)
**&nbsp;**		followed by sequencing using ZL1024
** Oligonucleotides used for generating *tsp-12 (5A mut)* using CRISPR-Cas9 **		
sgRNA (ZL1043)	ATCGGACATACTCGCTCAAC	
Repair template (ZL1042)	AGGTTCTCGGTATCTGTTTCGCTCAAAATCTCAAATCTGATGCTGCCGCCCAGCGTGCAAAAGCTGCCGCTACCCATTAATCAATCAAATAATCAATTAATCAACG	

## References

[R1] Barlowe C (2003). Signals for COPII-dependent export from the ER: what's the ticket out?. Trends in Cell Biology.

[R2] Bonifacino JS, Traub LM (2003). Signals for sorting of transmembrane proteins to endosomes and lysosomes.. Annu Rev Biochem.

[R3] Dancourt Julia, Barlowe Charles (2010). Protein Sorting Receptors in the Early Secretory Pathway. Annual Review of Biochemistry.

[R4] Dell'Angelica Esteban C., Bonifacino Juan S. (2019). Coatopathies: Genetic Disorders of Protein Coats. Annual Review of Cell and Developmental Biology.

[R5] Downes Katie W., Zanetti Giulia (2025). Mechanisms of COPII coat assembly and cargo recognition in the secretory pathway. Nature Reviews Molecular Cell Biology.

[R6] Dunn Cory D., Sulis Maria Luisa, Ferrando Adolfo A., Greenwald Iva (2010). A conserved tetraspanin subfamily promotes Notch signaling in
*Caenorhabditis elegans*
and in human cells. Proceedings of the National Academy of Sciences.

[R7] Farhan Hesso, Raote Ishier, Campelo Felix, Ge Liang, Hirschberg Koret, Forrester Alison, Zanetti Giulia, Lippincott-Schwartz Jennifer, Pastor-Pareja José Carlos, Perez Franck, Saito Kota, Malhotra Vivek (2025). Towards a unified framework for the function of endoplasmic reticulum exit sites. Nature Reviews Molecular Cell Biology.

[R8] Gomez-Navarro Natalia, Miller Elizabeth (2016). Protein sorting at the ER–Golgi interface. Journal of Cell Biology.

[R9] Hsu Victor W., Bai Ming, Li Jian (2012). Getting active: protein sorting in endocytic recycling. Nature Reviews Molecular Cell Biology.

[R10] Ichikawa K, Shoura MJ, Artiles KL, Jeong DE, Owa C, Kobayashi H, Suzuki Y, Kanamori M, Toyoshima Y, Iino Y, Rougvie AE, Wahba L, Fire AZ, Schwarz EM, Morishita S (2025). CGC1, a new reference genome for Caenorhabditis elegans.. Genome Res.

[R11] Kelly Bernard T, Owen David J (2011). Endocytic sorting of transmembrane protein cargo. Current Opinion in Cell Biology.

[R12] Liu Zhiyu, Shi Herong, Nzessi Anthony K., Norris Anne, Grant Barth D., Liu Jun (2020). Tetraspanins TSP-12 and TSP-14 function redundantly to regulate the trafficking of the type II BMP receptor in
*Caenorhabditis elegans*. Proceedings of the National Academy of Sciences.

[R13] Liu Z, Williams B, Wang L, Clark FK, Vignogna RC, Fromme JC, Liu J (2026). Tetraspanin TSP-12 and SUP-17/ADAM10 exhibit cell type-specific codependence for trafficking through the Golgi.. Proc Natl Acad Sci U S A.

[R14] Malhotra Vivek (2024). Tailored assemblies of COPII proteins in secretion. Journal of Cell Biology.

[R15] Sakaguchi Aisa, Sato Miyuki, Sato Katsuya, Gengyo-Ando Keiko, Yorimitsu Tomohiro, Nakai Junichi, Hara Taichi, Sato Ken, Sato Ken (2015). REI-1 Is a Guanine Nucleotide Exchange Factor Regulating RAB-11 Localization and Function in C.&nbsp;elegans Embryos. Developmental Cell.

[R16] Tu Linna, Tai William C. S., Chen Lu, Banfield David K. (2008). Signal-Mediated Dynamic Retention of Glycosyltransferases in the Golgi. Science.

[R17] Wang L, Liu Z, Shi H, Liu J (2017). Two Paralogous Tetraspanins TSP-12 and TSP-14 Function with the ADAM10 Metalloprotease SUP-17 to Promote BMP Signaling in Caenorhabditis elegans.. PLoS Genet.

[R18] Watson Emma T., Wegeng William R., Aravani Stamatina, Ernst Andreas M., von Blume Julia (2025). Mechanistic insights into cargo sorting and export from the Golgi apparatus. Nature Reviews Molecular Cell Biology.

